# Bioinformatic and experimental data pertaining to the role of the NLRP3 inflammasome in ovarian cancer

**DOI:** 10.1007/s00432-024-05988-9

**Published:** 2024-11-08

**Authors:** Ayisha A. Ashmore, Brinda Balasubramanian, Andrew Phillips, Viren Asher, Anish Bali, Paloma Ordóñez-Morán, Raheela Khan

**Affiliations:** 1 Derby Gynaecological Cancer Centre, Royal Derby Hospital, University Hospitals of Derby and Burton, Derby, UK; 2 Translational Medical Sciences Unit, School of Medicine, University of Nottingham, Nottingham, UK; 3 Translational Medical Sciences Unit, Biodiscovery Institute, Centre for Cancer Sciences, School of Medicine, University of Nottingham, Nottingham, UK

**Keywords:** NLRP3, Inflammasome, Ovarian cancer, Genes, Pyroptosis

## Abstract

The Nod-Like Receptor (NLR) family pyrin domain containing 3 (NLRP3) inflammasome plays a role in regulating inflammatory signaling and is a well-established contributor to pyroptotic cell death. It has been investigated extensively in cancer but there remains limited evidence of its role within ovarian cancer (OC). Bioinformatic investigation of gene expression data has highlighted that higher expression of NLRP3 and genes associated with the NLRP3 complex appear to be positively correlated with OC and may also have prognostic significance. However, heterogeneity exists within the results and experimental data is limited and contradictory. If the NLRP3 inflammasome is to be exploited as a therapeutic target, further laboratory-based investigation is required to determine its role in cancer. Furthermore, its relationship with clinically important characteristics such as histopathological subtype may be of key significance in developing targeted therapies towards specific cohorts of patients.

## Introduction

Ovarian cancer (OC) is the sixth most common cancer affecting women in the UK and accounts for 5% of all cancer deaths (“Ovarian cancer statistics,” 2015). 90% of OCs are epithelial in origin and present at an advanced stage resulting in poor survival outcomes (Cancer Research 2021; “Ovarian cancer statistics,” 2015). Unfortunately, gene-centric investigations focusing on specific mutations have been limited in describing tumour outcomes and have therefore been ineffective in the development of biomarkers or therapeutic targets. Additionally, many women are classified as developing OC *‘de novo’* (Kuo et al. 2009; Perets et al. 2013). As such, these tumours may not necessarily alter gene expression but instead interact with the tumour microenvironment (TME) facilitating tumour growth.

Chronic inflammation creates an environment that promotes cell proliferation, survival and genetic mutations, all of which can contribute to cancer development (Hanahan and Weinberg 2011). The production of reactive oxygen species (ROS) and reactive nitrogen species (RNS) resulting from higher metabolic activity in inflammatory environments directly damage deoxyribonucleic acid (DNA) (Liou and Storz 2010; Mantovani et al. 2008). Consequently, accumulated mutations can drive cancer transformation through intrinsic oncogene activation (Liou and Storz 2010; Mantovani et al. 2008). Additionally, chronic inflammatory environments can also activate signaling pathways such as nuclear factor kappa B (NF-kB), signal transducer and activator of transcription 3 (STAT3), and hypoxia-inducible factor 1-alpha (HIF-1α) which promote cell survival and proliferation in tumour cells (Mantovani et al. 2008). The resultant products, chemokines, cytokines and prostaglandins, have roles in leukocytic recruitment, inhibition of adaptive immunity, tissue remodeling and angiogenesis (Hanahan and Weinberg 2011; Mantovani et al. 2008; Wang and DuBois 2015; Zhao et al. 2021). This creates a cycle of inflammation within the TME which can further propagate tumour progression via epithelial-mesenchymal transition (EMT), invasion and metastasis (Hanahan and Weinberg 2011).

A key element of maintaining the inflammatory TME is through the recruitment and differentiation of immune cells. In chronic inflammation, the main infiltrating immune cells are macrophages and lymphocytes which invoke a number of mechanisms by which they contribute to a tumorigenic environment (Cai and Jin 2017; Zhao et al. 2021). For example, monocytes are recruited by tumour and stromal cells via various chemoattractant pathways before differentiating into macrophages (Mantovani et al. 2008; Yin et al. 2019). These circulating monocytes augment the population of tumour-associated macrophages (TAM) critical in the progression of inflammation in cancer (Yin et al. 2019). Once differentiated, the cells are able to polarize into the activated macrophage (M1) or the alternatively activated macrophage (M2) based on signaling pathways within the TME (Mantovani et al. 2008; Schweer et al. 2022; Yin et al. 2019). The M2 phenotype is thought to have tumorigenic properties as it promotes genetic instability, angiogenesis, tissue remodeling, and immunosuppression through its action on other immune cells and tumour cells (Mantovani et al. 2022; Yin et al. 2019). In OC, the most abundant immune cells present are TAMs exhibiting the M2 phenotype and their presence correlates with tumour progression (Colvin 2014). These findings also translate to survival data with patients expressing higher concentrations of M2 macrophages having poorer progression-free and overall survival (Lan et al. 2013).

A key common feature of myeloid cells is the presence of cytosolic, multiprotein complexes termed inflammasomes (Lillo and Saleh 2022). These proteins play a role in regulating the innate immune system and inflammatory signaling and are activated by pathogen or damage associated molecular patterns (PAMPS or DAMPs). Once activated, the inflammasome is instrumental in triggering the production of interleukin- 1β (IL-1β) and interleukin- 18 (IL-18) via caspase-1 leading to pyroptosis- a pro-inflammatory programmed cell death (Ke and Cai 2021; Kelley et al. 2019). In cancer, increased levels of DAMPs resulting from the combination of cellular stress (e.g. hypoxia, oxidative stress), sterile inflammation, uncontrolled cell proliferation and frequent cell death create and maintain a pro-inflammatory environment which likely drives conditions promoting tumour progression (Hernandez et al. 2016; Lillo and Saleh 2022).

The Nod-Like Receptor with a Pyrin Domain 3 (NLRP3) is one of the inflammasomes that has been implicated in tumour progression but its role in OC is not well understood. We argue that given the increased availability of DAMPs in the cancer TME, the NLRP3 inflammasome may mediate the propagation of the pro-inflammatory environment. This review aims to summarise probable genes linked to the NLRP3 inflammasome associated with OC to identify its role in tumour progression. Understanding these precise molecular mechanisms may help identify novel diagnostic, prognostic and therapeutic targets for OC.

## Mechanism of action of the NLRP3 inflammasome

The NLRP3 receptor resides within the cytoplasm in myeloid cells as inactive monomers prior to activation. It can be activated by multiple ligands in a ‘two-hit’ hypothesis with the first step requiring a priming signal mediated through the NF-κB pathway (Bauernfeind et al. 2009). This priming step can be regulated by several regulatory factors such as caspase-8 and mitochondrial reactive oxygen species (mtROS) (Juliana et al. 2012; Lemmers et al. 2007).

**Fig. 1:**
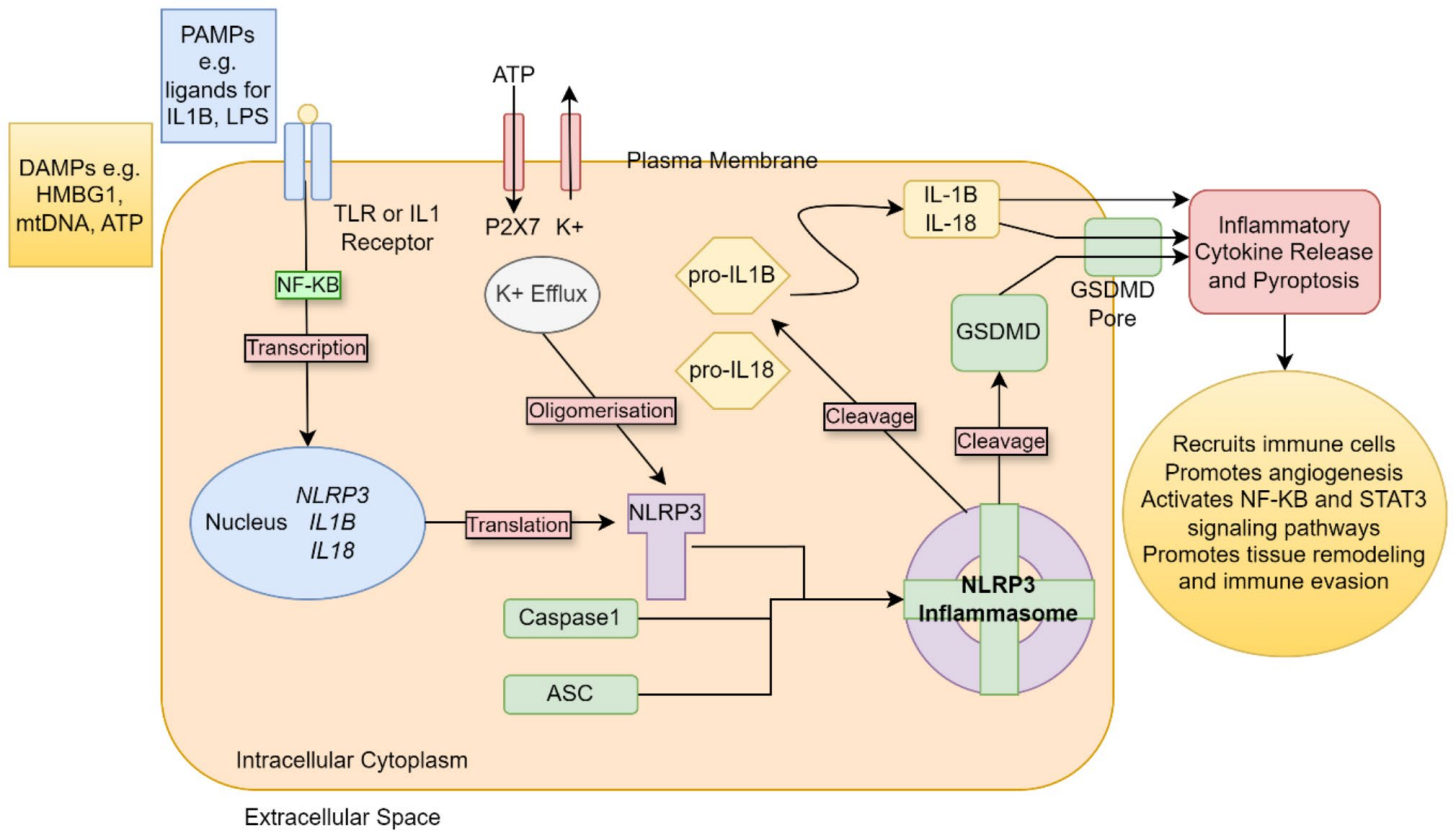
Mechanism of NLRP3 activation. Inflammatory stimuli such as PAMPs and DAMPS are sensed by receptors on the cell surface membrane. This causes upregulation of NLRP3 and IL1β transcription via the NF-κB pathway. Once translated, the NLRP3 components oligomerize in response to various cellular metabolic changes e.g. efflux of potassium ions. The NLRP3 inflammasome then assembles when the PYD of the NLRP3 binds with ASC. This then attracts procaspase-1 to form the inflammasome. Subsequently, procaspase-1 self cleaves into the active caspase-1 resulting in the cleavage of pro-IL-1β and pro-IL-18 into their active forms. Following the release of inflammatory cytokines, there is further inflammation resulting from additional recruitment of immune cells, with enhanced angiogenesis, tissue remodelling and activation of tumorigenic signalling pathways

Inflammatory stimuli including PAMPs and/or DAMPs trigger the first step of this pathway after detection by pattern recognition receptors (PRR) such as toll-like receptors (TLRs) or nod-like receptor 2(NOD2), tumour necrosis factor (TNF) receptor or IL-1 receptor, on the cell membrane (Bauernfeind et al. 2009; Sharma and Kanneganti 2021) (Fig. 1). PAMPs include ligands for IL-1βTNF and TLR causing nuclear translocation of NF-κB resulting in the upregulation of NLRP3 and IL-1β transcription e.g. lipopolysaccharides (LPS) (Hernandez et al. 2016; Sharma and Kanneganti 2021). DAMPs include extracellular molecules such as adenosine triphosphate (ATP), high mobility group box 1 (HMGB1), calreticulin, and uric acid amongst others (Conte et al. 2017; Hernandez et al. 2016; Martinon et al. 2006; Michaud et al. 2011; Obeid et al. 2007; Pellegatti et al. 2008; Scaffidi et al. 2002).

In OC, several studies have assessed the role of DAMPs in tumour progression and response to treatment. Singel et al. describe how mitochondrial DNA (mtDNA) could act as a potential DAMP to activate the NLRP3 inflammasome in advanced epithelial OC ascites (Singel et al. 2019). They report that patients with larger quantities of mtDNA in ascitic fluid had shorter progression free survival and thus a greater risk of disease progression within the first year of surgery. Indeed, mitochondrial dysfunction and subsequent mtDNA release is often accompanied by ROS generation and thus may be more prevalent in TME settings (Zheng et al. 2020). Similarly, HMBG1 has been reported as another DAMP involved in NLRP3 mediated tumour progression. Li et al. demonstrated higher levels of HMBG1 were found in patients with more advanced cancer in a study of 105 patients with epithelial OC (*p* < 0.0001) (Li et al. 2014).

The second step in NLRP3 activation is the assembly of inflammasome components via a number of pathways including efflux of potassium ions, calcium ions and chloride ions, lysosomal disruption, mitochondrial dysfunction, metabolic changes and trans-Golgi assembly (Swanson et al. 2019). NLRP3 activation then results in maturation and secretion of typically IL-1β but also IL-18. During assembly of the inflammasome, the NLRP3 monomer is activated by binding to the adaptor protein, apoptosis-associated speck-like protein containing a caspase-recruitment domain (ASC) resulting in oligomerization (Lillo and Saleh 2022). In this process, the pyrin domain (PYD) of the NLRP3 and the ASC bind together while the CARD domain of the ASC attracts procaspase-1 and forms the inflammasome (Lillo and Saleh 2022). Once activated, procaspase-1 autocatalyses into active caspase-1 (Sharma and Kanneganti 2021) and downstream processing of pro-IL-1β and pro-IL-18 into their smaller, active forms (Sharma and Kanneganti 2021). Additionally caspase-1 cleaves gasdermin-D which form pores on the plasma membrane through which IL-1β and IL-18 can be released and pyroptotic cell death can be actioned (Sharma and Kanneganti 2021).

Following activation of the P2X7 receptor, K^+^ exits from the cytoplasm resulting in the activation of inflammasome assembly (Muñoz-Planillo et al. 2013). High extracellular levels of ATP are a feature of the TME and thus their role as DAMPs via the purinergic P2X7 receptor is interesting to consider (Vultaggio-Poma et al. 2020). The P2X7 receptor is a transmembrane ion gated channel which is activated by its ligand, extracellular ATP (Lara et al. 2020). In a study of 11 OC patients and 2 OC cell lines, elevated expression of the P2X7 protein was discovered (Vázquez-Cuevas et al. 2014). Although activation of the receptor using ATP did not cause cell death, pharmacological antagonism with AZ10606120 and oxidized ATP resulted in reduced cell viability (Vázquez-Cuevas et al. 2014). Given higher metabolic activity producing extracellular ATP within the TME, this mechanism could further contribute to the cycle of self-propagating inflammation (Pellegatti et al. 2008).

Following pyroptosis, the released cytokines have a role in perpetuating the TME by recruiting and activating additional immune cells, promoting angiogenesis, activating pro-tumorigenic signaling pathways whilst also promoting tissue remodeling and immune evasion (Mantovani et al. 2008). For instance, IL-1β has been identified as an important trigger of OC ascitic vascular endothelial growth factor (VEGF) production, a key protein involved in tumour angiogenesis (Stadlmann et al. 2005). Similarly, IL-1 β has also been implicated in the activation of NF-κB pathways resulting in stromal fibroblast remodeling via a p53 knockdown (Schauer et al. 2013). Additionally, in endometriosis-associated ovarian carcinoma, increased of levels of IL-6 have been attributed to the NF-κB-IL-6-STAT3 signaling cascade (Browning et al. 2018; Leenen et al. 2021; Li et al. 2022b). IL-6 has long been associated with tumour growth through the activation of the STAT3 pathway. There is emerging evidence that tumour derived IL-1β drives the IL-6/STAT3 axis and thus may act as a master cytokine to increase other transcription factors (Tengesdal et al. 2021). In a study of melanoma cell lines, the investigators found that inhibiting NLRP3 using OLT1177, caused suppression of IL-1β mediated inflammation by disrupting the IL-1β/IL-6/STAT3 axis (Tengesdal et al. 2021). This resulted in reduced melanoma tumour progression (*p* < 0.01) (Tengesdal et al. 2021) and while not yet been observed in OC, it is possible that the NLRP3 inflammasome drives a complex pro-inflammatory and pro-tumour environment.

### Role of NLRP3 inflammasome in cancer

Whilst the NLRP3 inflammasome has not been investigated in OC, it has been implicated in numerous cancers (Dupaul-Chicoine et al. 2015; Ershaid et al. 2019; Wei et al. 2014; Zaki et al. 2010) (Table 1). In colorectal cancer (CRC), there is evidence of increased susceptibility of mice to develop CRC when IL-18 signaling is impaired (Dupaul-Chicoine et al. 2015; Zaki et al. 2010). Conversely, increased IL-1β production is implicated in promoting tumour growth of breast cancer cells (Ershaid et al. 2019). However, there is evidence suggesting a more dynamic role reporting low expression in normal hepatocytes, upregulation in hepatic inflammation and then downregulation in hepatocellular cancer (Wei et al. 2014).

**Table 1:** Examples of the role of the NLRP3 inflammasome in other cancers

Author	Finding	Type of cancer
Dupaul-Chicoine et al. (2015)	Protector	Colorectal Cancer
Zaki et al. (2010)	Protector	Colorectal Cancer
Ershaid et al. (2019)	Exacerbator	Breast Cancer
Wei et al. (2014)	Protector but more dynamic relationship	Hepatocellular Cancer

### NLRP3 and genes related to NLRP3 in OC

Gene expression of the NLRP3 inflammasome pathway in OC has been explored through a variety of bioinformatic studies. Bioinformatic analysis of messenger ribonucleic acid (mRNA) expression data demonstrated upregulation of the NLRP3 gene in 381 OC cases when compared to 71 normal tissue samples from The Cancer Genome Atlas (TCGA) databases (Li et al. 2023). Increasing expression levels were also reported between stages, with stages 3 and 4 showing higher NLRP3 expression. Similarly, poorer survival probability was noted with higher NLRP3 expression suggesting that the NLRP3 gene may act as an independent prognostic gene in OC.

Differing results were found from primary analysis of human and chicken OC tissue. Luborsky et al. reported no significant differences in mRNA expression of NLRP3, caspase-8 and caspase-11 between tumour containing and non-tumour containing ovaries in both human and chicken OCs (Luborsky et al. 2020). However, mRNA expression was higher for hallmarks of inflammasome activity which included caspase-1, IL-β and IL-18 leading the authors to conclude that inflammasome expression is associated with OC but the nod-like receptor (NLR) sensor type could not be determined (Luborsky et al. 2020). The same study also examined the role of the aryl hydrocarbon receptor (AHR) which has been detailed to be a potential tumour suppressor and NLRP3 regulator. AHR negatively regulates NRLP3 transcription and interacts with its repressor to create a feedback cycle of promotion and inhibition (Huai et al. 2014; Vogel and Haarmann-Stemmann 2017). Luborsky et al. discovered that the AHR repressor was significantly upregulated in human OC suggesting that NLRP3 transcription is downregulated in OC and thus lower expression could result in tumour progression (Luborsky et al. 2020). However, the authors only included 3 serous OCs when assessing mRNA expression and did not perform subgroup analysis based upon histological subtype or stage of cancer and so conclusions must be limited.

Other genes have also been implicated in the NLRP3 inflammasome pathway (Table 2). A pan cancer bioinformatic analysis of the NLRP3 inflammasome, which did not include OC, identified 30 genes potentially associated with NLRP3 inflammasome expression. C (Ju et al. 2020). Similar genes have been described in further bioinformatic analysis of pyroptosis genes in OC. Ye et al. first identified 31 differentially expressed genes in OC of which NLRP3, IL1-β, IL-18, CASP8, TNF, AIM2, PYCARD, GSDMA and GSDMC were all enriched suggesting NLRP3 inflammasome activity (Ye et al. 2021). Wu et al. also reported differentially expressed genes in OC in a bioinformatic analysis of pyroptosis-related genes (Wu et al. 2023). They noted an upregulation of CASP8, IL-1β, IL-18, GSDMA, GSDMC, and PYCARD, as well as downregulation of PLCG1. Although the authors did not find significant change in the NLRP3 gene expression, the associated genes and gene products had a protein-to-protein interaction (PPI) score of 0.9 indicating confidence in their interactions. This may suggest that the NLRP3 inflammasome pathway is active and that pyroptosis occurs within OC cells but the effect of the NLRP3 on clinical tumour initiation or progression cannot be determined by this type of analysis alone.

**Table 2:** List of gene names and function related to the NLRP3 inflammasome

Gene	Name	Protein function
AIM2	Absent in melanoma 2	Cytoplasmic sensor found in T cells that recognises presence of double stranded DNA. Activated AIM2 recruits ASC resulting in caspase-1 binding and formation of AIM2 inflammasome.
CASP1	Caspase-1, apoptosis-related cysteine peptidase	Enzyme that binds with ASC and activates to cleave inflammatory cytokines e.g. pro-IL1β and IL-18. Also cleaves gasdermin D into active mature peptide.
CASP3	Caspase-3, apoptosis-related cysteine peptidase	Enzyme that interacts with other caspases resulting in the activation of caspase-6. Is activated by interaction with caspase-8.
CASP6	Caspase-6, apoptosis-related cysteine peptidase	Enzyme that interacts with other caspases causing downstream activation of other caspase family enzymes.
CASP8	Caspase-8, apoptosis-related cysteine peptidase	Enzyme that interacts with other caspases causing downstream activation and modification of the NF-κB pathway.
ELANE	Neutrophil elastase	Proteinase which is secreted by neutrophils during inflammation, localises to neutrophil extracellular traps (NETs).
GSDMA	Gasdermin family, A	Substrate of inflammatory caspases.
GSDMC	Gasdermin family, C	Substrate of inflammatory caspases.
IL-1β	Interleukin-1 family, beta	Cytokine protein activated by caspase-1.
IL-18	Interleukin-18 family	Cytokine protein activated by caspase-1.
NLRP3	Nod-Like Receptor family, pyrin domain containing 3	Nod-like receptor family domain containing protein 4 and key component of NLRP3 inflammasome.
NLRC	Nod-Like Receptor family, containing Caspase activation and recruitment domain (CARD)	Nod-like receptor family CARD domain containing protein 4.
PJVK	Gasdermin family, Pejvakin	Protein member of gasdermin family.
PLCG1	Phospholipase C, gamma 1	Cell growth factor protein involved in apoptosis.
PYCARD	Pyrin domain (PYD) and CARD domain containing	Also known as apoptosis associated speck-like protein containing a CARD. Binds to NLRP and pro-caspase 1 to assemble inflammasome.
TNF	Tumour necrosis factor	Cytokine protein which responds to IL-1 signaling.

On further interrogation of public datasets, several studies have identified ‘hub’ genes that are correlated with a number of other genes which are upregulated in OC and thus may be related to tumorigenesis and progression. Both NLRP3 and PLCG1 have been identified as such genes in a study of 379 OC tumour samples (Liu et al. 2022b). The study reports that NLRP3 was correlated with 24 genes but highly correlated with NLRC4, IL-1β and CASP1. Specifically, NLRP3 was positively correlated with genes which were upregulated in OC whereas PLCG1 was negatively correlated in genes which were upregulated in OC such as CASP1, PYCARD and IL-18. This confirmed that NLRP3 was higher in OC consistent with previously published literature.

Similarly, a bioinformatic analysis of necroptosis-related genes identified CASP8 as a potential hub gene in the tumorigenesis and development of OC (Wang et al. 2022). The authors identified that it was consistently upregulated in OC tissue and PPI analysis demonstrated multiple connections with other genes within the necroptosis pathway (Wang et al. 2022). Similar findings were noted by Zhang et al. who also reported that CASP8, IL-1β, IL-18, and TNF may be ‘hub’ genes within a pyroptosis pathway in OC (Zhang et al. 2023).

Clinical correlation with gene expression is an important tool for understanding the utility of these genes in clinical practice. A number of studies have developed prognostic models to predict survival and progression of OC based on the presence or absence of certain genes (Liu et al. 2022b; Wang et al. 2022; Ye et al. 2021; Zhang et al. 2023; Zhou et al. 2021). Two studies included genes that are suspected to have a role in the NLRP3 inflammasome pathway within their prognostic models. Ye et al. identified that AIM2, ELANE, PJVK, CASP3, CASP6 and GSDMA expression levels could be utilized to calculate a risk score for patients with OC. They then categorized patients into high and low-risk groups reporting that patients in the high-risk group had more deaths and shorter survival times than those in the low-risk group. Receiver operating characteristic (ROC) analysis was also conducted to assess the sensitivity and specificity of the model. The area under the curve (AUC) ranged from 0.628 to 0.607 between 1 and 3 year survival meaning the model was able to successfully discriminate between groups in relation to survival (Ye et al. 2021).

Zhang et al. developed a similar model using AIM2, CASP3, CASP8, DHX9, GSDMA, GZMB, NLRP3, NLRP6 and ZBP1 expression levels. The study again classified patients into high and low-risk groups and found that those with a high-risk score had a higher risk of death and a shorter survival time. ROC analysis found an AUC of between 0.671 and 0.535 for survival times of 1–3 years (Zhang et al. 2023). Additionally they found that the expression of NLRP3, NLRP6 and GSDMA were higher in patients within the high-risk group thus implicating the role of the NLRP3 inflammasome pathway in survival (Zhang et al. 2023). Critically, the authors also reported that AIM2, CASP3, CASP8 and ZBP1 expression were significantly negatively correlated with advancing clinical stages (*p* < 0.05) (Zhang et al. 2023). As caspase-8, the gene product of CASP8, is known to regulate NLRP3 inflammasome activity, it could be hypothesized that a reduction in its expression could lead to dysregulation of the NLRP3 inflammasome resulting in OC progression.

The AIM2 (Absent In Melanoma 2) gene has repeatedly been identified as an important gene related to the NLRP3 inflammasome. The AIM2 protein is another form of cytoplasmic sensor which fulfills a similar role to NOD-like receptors within inflammasome complex. Although it forms its own inflammasome, there is evidence that it interacts with the NLRP3 and could thus lead to NLRP3 inflammasome activation (Chang et al. 2017). It was found that both NLRP3 and AIM2 were significantly associated with statistically significant poor prognosis in patients with endometriosis-associated OC. On further immunohistochemical testing, AIM2 proteins were found in higher abundance within endometrioid and clear cell carcinomas of the ovary suggesting its involvement in malignant transformation (Chang et al. 2017). Further studies have associated AIM2 expression with resistance to bevacizumab and shorter progression free survival (Hsu et al. 2021). However further in vivo study of the NLRP3 and AIM2 interaction in carcinogenesis is yet to be performed.

Similarly, TRPM2 (transient receptor potential melastatin 2) has been implicated in NLRP3 inflammasome mediated pyroptosis (Huang et al. 2023). TRPM2 is a non-selective cationic channel which has been associated with cell apoptosis (Zhang et al. n.d.). Bioinformatic analysis of TCGA, Gene Expression Omnibus (GEO) and the Genotype-Tissue Expression (GTEx) databases along with primary tissue immunohistochemistry showed high expression in OC which was related to poorer prognosis. Further investigation using real-time quantitative polymerase chain reaction (PCR) revealed a strong correlation with NLRP3 suggesting an interaction between TRPM2 and the NLRP3 inflammasome in OC progression (Huang et al. 2023).

Another gene that has been implicated in inflammasome activity include FOSL (fos-like antigen 2) (Li et al. 2022a). FOSL is a member of the activator protein-1 transcription family which regulates growth, development and immune response (Li et al. 2018). Its expression in OC cells was established using quantitative PCR which showed significantly higher expression in cancer tissue compared with adjacent tissue (Li et al. 2022a). Furthermore, when FOSL was overexpressed, Western blotting demonstrated that inflammasome-associated proteins such as caspase-1, IL-1β and IL-18 were downregulated and apoptosis was significantly decreased (Li et al. 2022a). Although the NLRP3 inflammasome was not expressly investigated, these findings suggest its activity could be involved within OC.

### NLRP3 and long non-coding RNA

In addition to genes, long non-coding RNAs (lncRNA) have been identified as potential markers of inflammasome activity in OC. HOTTIP is a lncRNA which has been found to upregulated in clinical tissues and cell lines of OC (Tan et al. 2021). When HOTTIP was silenced using small interfering RNA targeting HOTTIP (si-HOTTIP), there was significantly increased cell proliferation, activation of caspase-1 and increased expression levels of IL-1β, IL-18, and NLRP1 (Tan et al. 2021). Although this study did not expressly investigate the NLRP3 inflammasome, it does suggest a possible role through the upregulation of NLRP3 inflammasome pathway products with HOTTIP acting as a pyroptosis suppressor and potential oncogene in OC progression.

Prognostic models have also been developed for lncRNAs in OC. Cao et al. developed a gene signature based on 8 lncRNAs (DICER1-AS1, MIR600HG, AC083880.1, AC109322.1, AC007991.4, IL6R-AS1, AL365361.1, and AC022098.2) and found shorter survival in patients classified as high risk (Cao et al. 2022). KEGG enrichment analysis showed that mRNA associated with pyroptosis-related lncRNAs was enriched in the NOD-like receptor signaling pathway (Cao et al. 2022).

### Epigenetic modification of NLRP3

Epigenetic modification of gene function has been briefly described within the context of inflammasomes and OC. It has been postulated that silencing of the ASC gene, a core component of the NLRP3 inflammasome, may have a role in cancer cell survival. Terasawa et al. described that silencing the ASC gene by DNA methylation and histone deacetylation can promote cancer cell survival. Thus, inhibiting these functions could restore ASC expression and consequently lead to apoptosis of ovarian clear cell cancer cells (Terasawa et al. 2004). Contrarily, this would suggest that the NLRP3 inflammasome has a protective effect and adds to the discussion around its role.

MicroRNAs (miRNA) may act as epigenetic modulators of gene expression. The relationship between the NLRP3 inflammasome and microRNAs has been investigated in one study. Wu et al. showed that NLRP3 was overexpressed in OC cells in contrast to poor expression of microRNA-22 (miR-22) (Wu et al. 2021). The study demonstrated that NLRP3 was the target gene for miR-22 via luciferase activity assays and so altering miR-22 levels in OC cell lines could regulate NLRP3 expression (Wu et al. 2021). Hereby, low miR-22 levels were associated with high NLRP3 expression and cell proliferation and thus poor prognosis (Wu et al. 2021) implicating NLRP3 activity with poor outcomes in OC (See Table 3).

**Table 3:** Summarises the findings from NLRP3 and related gene expression in identified bioinformatic studies

Author	Bioinformatic Tools	Finding	NLRP3 expression Protector or Exacerbator in OC?
Li et al. (2023)	TCGA Database	Upregulation of NLRP3 expression in OC samples compared with normal tissue.	Exacerbator
	GEO Database		
	GEO2R Tool		
	R package software		
Ye at al. (2021)	TCGA Database	Upregulation of NLRP3 expression in OC compared with normal tissue.	Exacerbator
	GTex Database		
		IL1-β, IL-18, CASP8, TNF, AIM2, PYCARD, GSDMA and GSDMC also upregulated suggesting NLRP3 inflammasome activity in OC.	
Wu et al. (2023)	TCGA Database	No significant difference in NLRP3 expression between OC and normal tissue	No difference
	GTex Database		
	STRING software		
	R package software		
		CASP8, IL-1β, IL-18, GSDMA, GSDMC, and PYCARD upregulated in OC and downregulation of PLCG1 suggesting NLRP3 inflammasome activity.	
Liu et al. (2022b)	TCGA Database	NLRP3 and PLCG1 identified as hub genes in OC samples.	Exacerbator
	GTex Database		
	GEO Database		
		NLRP3 was correlated with 24 genes but highly correlated with NLRC4, IL-1β and CASP1. NLRP3 positively correlated with genes upregulated in OC. PLCG1 negatively correlated with genes upregulated in OC.	
	R package software		
Zhang et al. (2023)	TCGA Database	CASP8, IL-1β, IL-18, and TNF identified as potential hub genes in OC.	Exacerbator
	GTex Database		
	GEO Database		
	GeneCards database	NLRP3 expression higher in patients within high-risk groups when classified using gene risk model.	
	R package software		
	STRING software		
	Cytoscape software		
Chang et al. (2017)	GO database and tool	NLRP3 significantly associated with poor prognosis in patients with endometriosis-related OC.	Exacerbator
Huang et al. (2023)	TCGA Database	TRPM2 expression associated with poorer prognosis in OC. TRPM2 strongly correlates with NLRP3.	Exacerbator
	GTEx Database		

Abbreviations: The Cancer Genome Atlas (TCGA), Genotype-Tissue Expression project (GTex), Gene Expression Omnibus (GEO), Gene Ontology (GO)

### Correlation of NLRP3 gene expression with experimental data

This review has identified higher expression of NLRP3 genes in OC when compared with normal ovarian tissue in bioinformatic data of gene expression. There is also higher mRNA expression of associated genes in OC including CASP8, IL1B, IL18, GSDMA, GSDMC and PYCARD, with several associated with poorer survival. This corroborates higher NLRP3 expression with poorer OC outcomes. Other gene interactions could regulate the NLRP3 inflammasome activity including AIM2, TRPM2, CASP8. The expression of these genes is also related to poorer prognosis in OC. However, there are limitations to these bioinformatic methods due to sample quality and storage, use of appropriate control samples and an inability to always stratify for clinically relevant groups such as histopathological subtype or stage of disease. As such it is important to correlate bioinformatic data with experimental data taking into consideration clinical characteristics. Table 4 summarises experimental studies which investigate gene expression of NLRP3 inflammasome in OC and has built upon findings from Lui et al. (C. Liu et al. 2022a).

Despite evidence of increased mRNA and gene expression of NLRP3, little translational evidence exists to which these findings can be correlated. In a study of 45 patients with epithelial OC, caspase-8 gene expression levels were 25-times higher in metastatic epithelial ovarian samples compared with benign ovarian tissue or primary epithelial OC (Braga et al. 2014). This suggested inflammasome activation could be associated with more severe disease. However, the authors also concluded that caspase-8 can induce non-apoptotic pathways and so higher levels could be a result of higher levels of active proteins which can reduce apoptotic activity in addition to causing dysregulated proliferation of cells (Braga et al. 2014). These features are also characteristic of metastatic ovarian tumours (Braga et al. 2014).

Li et al. describe the expression of IL-1α and IL-1β genes in the ascitic fluid of 4 epithelial OC patients and three OC cell lines (Li et al. 1992). Keita et al. reported significant expression of IL-1α in clear cell OC cells whilst high expression of IL-1β was seen in endometroid and serous cancer cell lines (Keita et al. 2010). Both studies suggest higher expression of these cytokines is associated with pathological processes and thus the presence of the NLRP3 inflammasome confers greater risk.

Gasdermins are members of pore-forming effector protein which cause pyroptotic cell death when inserted into cell membranes (Broz et al. 2020). They are activated by cleavage of the C-terminal domain from the cytotoxic pore forming N-terminal domain by caspases which are in turn, activated by the assembly of the NLRP3 inflammasome (He et al. 2015; Shi et al. 2015). Their presence can therefore be indicative of inflammasome activity but may not be specific to the NLRP3 inflammasome. There is a variation in findings into the role of gasdermin C and gasdermin D. Liu et al. report that GSDMC (gene for gasdermin C) is downregulated (Liu et al. 2022b), but this is in discordance with five other studies showing upregulation (Berkel and Cacan 2021; Cao et al. 2022; Wu et al. 2023; Ye et al. 2021; Zhang et al. 2023). In experimental data, Berkel and Cacan found that there was also variation in gasdermin expression between histological subtypes of OC (Berkel and Cacan 2021). Gasdermin B was increased in mucinous OCs compared with serous or endometrioid subtypes (Berkel and Cacan 2021). Similarly, gasdermin D was elevated in clear cell and serous subtypes as compared with endometrioid subtypes. However, it appears that high expression of GSDMC and GSDMD are associated with shorter progression free survival in TP53 mutated OC with between 5 and 12 month differences in survival between high and low expression levels (Berkel and Cacan 2021). These findings suggest the increased activity of the NLRP3 inflammasome in OC via gasdermin mediated pyroptosis but the association is yet to be fully explored. It could be speculated that the differences in gene expression could be related to clinical factors such as age, histological subtype and stage of the tumours investigated. However, these clinically relevant details have not been correlated with expression outcomes in all studies and therefore may be worth investigating further if novel therapeutic targets are to be developed.

**Table 4:** Summary of NLRP findings from experimental studies from RNA sequencing data

Author	Finding	NLRP3 expression Protector or Exacerbator in OC?
Luborsky et al. (2020)	No significant difference in NLRP3 expression between tumour and non-tumour containing ovaries of human and chicken OC.	No difference
Li et al. (2022b)	FOSL expression higher in OC than adjacent tissue. FOSL overexpression associated with reduction in inflammasome-related proteins e.g. caspase-1, IL-1β and IL-18.	Protector
Tan et al. (2021)	HOTTIP lncRNA silencing in OC associated with increased cell proliferation and increased expression of caspase-1, IL-1β and IL-18 suggesting NLRP3 involvement in cell proliferation.	Exacerbator
Terasawa et al. (2004)	Silencing ASC (core component of NLRP3) causes cancer cell survival. Restoring ASC expression led to apoptosis of ovarian clear cell cancer cells.	Protector
Wu et al. (2021)	NLRP3 overexpressed in OC cell lines.	Exacerbator
Braga et al. (2014)	Caspase-8 levels 25 times higher in metastatic epithelial OC samples compared with benign ovarian tissue or primary epithelial OC.	Exacerbator
Li et al. (1992)	Expression of IL-1α and IL-1β in OC cell lines and ascites of OC patients.	Exacerbator
Keita et al. (2010)	High expression of IL-1β in endometrioid and serous cancer cells lines.	Exacerbator
Berkel and Cacan (2021)	Higher expression of gasdermin C and D in TP53 mutated OC associated with shorter progression free survival.	Exacerbator

### Association of NLRP3 with chemoresistance in OC

Although experimental evidence seems to overwhelmingly support the upregulation of the NLRP3 in OC and its association with poorer outcomes, there is also some evidence that NLRP3 expression may have anti-tumour properties. Heath et al. investigated the role of the NLRP3 inflammasome in TAMs in omental biopsies of high grade serous OCs (Heath et al. 2021). They found that in TAMs treated with carboplatin, there was evidence of increased NLRP3 inflammasome activation demonstrating a phenotypic change to promote anti-tumour activity (Heath et al. 2021). Clinically this effect is important due to the development of treatment resistance in patients undergoing chemotherapy. New therapies could target TAMs to cause a phenotypic alteration and enhance the chemotherapy-induced immune response (e.g. by activating the NLRP3 inflammasome) (Heath et al. 2021). This could not only improve disease free survival but could also reduce the dosage of chemotherapy required and thus limit treatment resistance (Heath et al. 2021).

Similarly, Lau et al. investigated the role of paclitaxel in OC chemotherapy (Lau et al. 2020). Anthracyclines causes translocation of endoplasmic reticulum chaperones such as calreticulin to the cell surface of tumour cells. These are subsequently recognised by dendritic cells resulting in phagocytosis (Lau et al. 2020). The resultant release of DAMPs such as ATP can lead to NLRP3 inflammasome activation and promote downstream anti-tumour activity in both murine and human OC cells in vitro (Lau et al. 2020). Furthermore, paclitaxel also activated the NF-κB signaling pathway which could be another route into upregulating NLRP3 inflammasome expression (Lau et al. 2020).

### Therapeutic potential of exploiting inflammasome related pathways

Whilst the expression and function of NLRP3 in OC is important to understand, its ability to be exploited as a therapeutic target will have significant clinical relevance. There have been no studies to assess whether disrupting NLRP3 related pathways can cause tumour regression in OC. However, there have been studies in other types of cancers which may highlight the benefits of pursuing further investigation in OC.

In CRC, a phase 2 trial of 32 patients with metastatic CRC which was refractory to chemotherapy and anti-angiogenic therapy was conducted (Isambert et al. 2018). The patients were administered anakinra in combination with 5-FU and bevacizumab. Anakinra is a recombinant form of IL-1 receptor antagonist which has downstream effects on NLRP3 inflammasome activation. Median progression free survival was reported at 5.4 months (95% CI 3.6–6.6) and overall survival of 14.5 months (95% CI 9-20.6) which was superior to other third line therapies without an NLRP3 inflammasome pathway disruptor.

Cankinumab is an anti-IL-1β monoclonal antibody. The Canakinumab Anti-inflammatory Thrombosis Outcomes Study (CANTOS) demonstrated a significant reduction in lung cancer-caused mortality in a trial of 10,000 randomised patients (Ridker et al. 2017). Results from phase 1 and 2 trials for a competitor, nadunolimab, in melanoma, pancreatic and triple negative breast cancer are awaited (“Canakinumab’s cancer career ends with a whimper,” 2022; “Cantargia advances TRIFOUR trial to randomized stage following promising early safety and efficacy of nadunolimab in triple-negative breast cancer - Cantargia,” n.d.).

Some molecules have been developed which directly target components of the inflammasome itself. For example, Tranilast (N-[3,4-dimethoxycinnamoyl] anthranilic acid) directly blocks the NACHT domain of the NLRP3 inflammasome and thus prevents its assembly (Huang et al. 2018). In colorectal and lung cancer cells lines Tranilast was demonstrated to inhibit tumour growth by way of increasing apoptotic activity (Lo Re et al. 2018). MCC950 is a NLRP3 inflammasome inhibitor which has been explored in head and neck squamous cell cancer. Chen et al. report that the molecule was able to reduce IL-1β production which also resulted in human cancer cell lines but also delayed tumorigenesis in a mouse model (Chen et al. 2018). Similar findings were reported in pancreatic cancer showing that MCC950 was able to reduce the cell viability of pancreatic cancer cells (Yaw et al. 2020). Further work is needed on a larger scale to determine whether MCC950 is a viable and safe drug in humans. Another promising molecule named OLT1177 is an active β-sulfonyl nitrile molecule. In vitro, it has reduced IL-1β and caspase-1 activity in human blood neutrophils (Marchetti et al. 2018). However, there have been no studies on cancer cell lines and thus its effectiveness as a cancer therapeutic target remains to be seen.

## Conclusion

This is the first review to synthesise the bioinformatic studies that have focused on the NLRP3 inflammasome and OC whilst correlating findings with experimental data. In summary there appears to be higher expression of NLRP3 and related genes associated with OC. However, the relationship between expression and clinical characteristics such as histopathological subtype and stage have not been fully explored. There may also be prognostic significance to NLRP3 expression with some studies identifying NLRP3 is associated with poorer survival. Experimental data correlating these findings are limited and have shown heterogeneous results.

Further experimental work is required to elucidate NLRP3 expression in OC and whether this translates into protein expression with functional changes. In this context, characterizing the TME in terms of its inflammatory profile in OC could further clarify the mechanism by which the disease may progress. In doing so, it would be hoped that advances may be made in developing a viable drug target by altering the NLRP3 inflammasome pathway. However, more importantly, clinical correlation with histopathological subtypes, stages of disease and the use of chemotherapy are crucial in determining whether this is a feasible therapeutic approach.

## Data Availability

No datasets were generated or analysed during the current study.
